# Development and validation of a new instrument to measure social pain

**DOI:** 10.1038/s41598-021-87351-3

**Published:** 2021-04-15

**Authors:** Ulrich Stangier, Johanna Schüller, Elmar Brähler

**Affiliations:** 1 Department of Clinical Psychology and Psychotherapy, Goethe-University, Frankfurt, Germany; 2 Department for Psychosomatic Medicine and Psychotherapy, University Medical Center of Johannes Gutenberg University of Mainz, Mainz, Germany

**Keywords:** Psychology, Social behaviour

## Abstract

Social pain is an emotional reaction to social exclusion which has been widely investigated in experimental settings. We developed the Social Pain Questionnaire (SPQ) and examined its factor structure, reliability, and construct validity. We constructed a 46-item pool that covered a broad range of situations related to social pain. Using three different subsamples (Online convenience sample: *n* = 623, Representative sample: *n* = 2531, Clinical sample of outpatients seeking psychotherapy: *n* = 270) we reduced the item pool to 10 items for the final SPQ scale, paying particular attention to content validity and factorial structure. Convergent, divergent and discriminant validity were assessed using standardized measures of related constructs and group differences. For the final 10-item version, a good factorial structure and reliability were found. Convergent validity was supported by correlations with related instruments of interpersonal sensitivity, attachment styles, depression and social anxiety. The representative and clinical sample differed significantly in social pain. The SPQ is an economic self-report measure with solid psychometric properties. Our data support the factorial, construct and convergent validity. The SPQ can be used to clarify the role of social pain in mental disorders and to incorporate interventions targeted towards social pain in psychotherapeutic settings.

## Introduction

Social pain has been defined as a negative emotional state triggered by the perception of being excluded or rejected from a group or a relationship to a significant person^1^. However, the construct has been conceptualized in different ways concerning triggers, quality of emotional states and psychological mechanisms involved^2^.

The feeling of “hurt”^3^ is a negative emotional state, which is different from other social emotions such as fear, shame or guilt. Due to the subjective overlap with physical pain concerning intensity and adversity, as well as similarities in the neurobiological processing^4^, the term “social pain” has become more common in literature. The feeling of being hurt has been conceptualized as a reaction to interpersonal rejection, associated with a relational devaluation^5^. In contrast, social pain has been more often used in the context of social exclusion and ostracism, to characterize the target’s reaction to threats for the need for belonging^1,4^. Both rejection and exclusion are not restricted to specific social domains but may occur in romantic relationships, kin and family, friends, peers, or groups^6^.

The social-physical pain overlap theory^4^ suggests that both systems share common physiological mechanisms because evolutionarily, rejection and social exclusion posed similarly serious threats to survival as physical injury. However, this theory has been criticized, since data from fMRI studies show inconsistent results with regard to the activation patterns of the brain involved in social pain, and also indicate that this pattern is neither specific for social pain nor for pain^7^. Besides the controversy about the neurobiological basis for social pain, however, there is consensus that the cyberball paradigm which has been used in most of these studies is a reliable and valid experimental paradigm to induce social pain as a reaction to social exclusion^8^.

Extreme sensitivity to rejection, relational devaluation and perceived social exclusion are included in the criteria for several psychiatric diagnoses, especially social anxiety disorder (SAD), depression and borderline personality disorder (BPD)^9^. Furthermore, early emotional neglect, abuse, and chronic rejection during childhood are important risk factors for the later development of these disorders^10^. Research focusing on the relationship of social pain to psychopathology focused mainly on two methodological approaches. Using self-report measures, significantly increased fear of rejection was found in patients with depression, personality disorders, and SAD^11^, among the personality disorders most prominently BPD^12^. Interestingly, using the cyberball paradigm, a similar pattern evolved for diagnoses with high ratings of need threat^13^: patients with BPD and chronic depression^14,15^, as well as SAD^16^, but not posttraumatic stress disorder^17^ showed significantly more fear of rejection than healthy controls.

However, methodological problems limit the interpretation of these results. In the cyberball experiment, assessment of social pain is based on the needs-threat scale^18^. The needs-threat model^19^ assumes that social exclusion threatens four fundamental needs: belonging, self-esteem, control and meaningful existence. However, construct validity has been questioned due to high intercorrelations and inconsistent correlations with other questionnaires assessing diverging needs^20^. Furthermore, it should be noted that the need-threat scale is related to short-term emotional reactions to the experience of being excluded in the cyberball game, thus covering rather a response to a specific situation than a consistent response tendency to perceive social pain in different situations.

Among standardized self-report instruments, the Adult Rejection Sensitivity Questionnaire (ARSQ)^21^ refers to anxious expectations in situations, in which the rejection of a request by significant others is anticipated, but not actually experienced. The Interpersonal Sensitivity Measure (IPSM)^22^ focuses on excessive awareness and vigilance of the behavior and feelings of others, particularly on perceived or actual criticism or rejection. However, the theoretical basis of this measure refers to the concept of ‘depression-prone’ personality, but is also derived from the clinical experience of the authors. The items of the IPSM assess emotional reactions to a broad range of interpersonal situations, covering not only fear of rejection but also susceptibility to critique, self-criticism, anxiety, shyness and dependency. In addition, besides the unclear theoretical relationship to social pain, subsequent studies did not support the original dimensional structure either of the ARSQ^23^ or the IPSM^24^. Finally, the Hurt Feelings Scale by Leary and Springer^3,25^ is a six-item scale referring to hurt as specific emotion triggered by interpersonal rejection, developed based on qualitative interviews on emotional states following hurtful episodes. Although the Hurt Feelings Scale is more specifically related to the emotional components of social pain than the other instruments mentioned before, its psychometric properties have not been evaluated systematically. The items have been derived from content analyses of qualitative interviews referring to interpersonal triggers in daily life, but a modification referring to Hurt Feeling Proneness has not been validated to our knowledge. To conclude, there is a lack of standardized and validated instruments specifically related to the conceptual framework of perceived social pain as a response predisposition, which are appropriate also for clinical settings.

This article describes the development and validation of a new scale focusing on the assessment of social pain. We present data from three substudies:

1. Generation of an initial item pool and assessment of psychometric criteria in a self-selected online sample.
2. Two-step reduction to 10 items, using a genetic algorithm.
3. Assessment of the factorial structure, and the convergent, divergent and discriminant validity of the final scale.

## Results

### Substudy 1: development of an item pool

The theoretical basis for the construction of an item pool for the Social Pain Questionnaire (SPQ) was to cover different emotional and embodied cognitive responses to interpersonal situations related to social exclusion and interpersonal rejection. The item pool contained 46 statements referring to diverse everyday interpersonal situations related to rejection or exclusion with friends, romantic partners, family, acquaintances, peer groups and professional relationships (31 items), as well as unspecified situations with others (15 items). The responses described in the statements were either related to emotional responses to others’ behaviors (15 items), embodied cognitions/interpretation of the others’ behaviors (14 items), or both (17 items). Based on entries in the most popular German Thesaurus (www.duden.de), synonyms were used for social or mental pain in German language, such as “hurt” (“verletzt”), “offended” (“gekränkt”), “rejected” (“zurückgewiesen”) or “feel affected” (“weh tun”). The items are evaluated on a five-point Likert scale ranging from 0 = “Applies not at all to me” to 4 = “Applies exactly to me” Although intensity ratings are more common in emotion research, appropriateness appears to be more suitable for the judgement of a complex interaction of situation, emotional response and interpretation of the behavior of others. To form the total score, the mean of all item scores is calculated. This preliminary item pool was presented to a self-recruited online sample for a first assessment of the factorial structure. The model fit in the complete item pool was poor (${\chi }^{2}$ = 5071.146, p < 0.001; CFI = 0.671, RMSEA = 0.085, SRMR = 0.071), indicating levels of misspecification. Modification indices showed several error covariances, indicating problems with the unidimensionality of the instrument. In the second substudy, we, therefore, reassessed the item pool, to improve the construct definition and model fit.

### Substudy 2: item reduction

In the first step, a preliminary reduction was performed to minimize redundancy and clarify construct specification within the item pool. The intermediate version was then presented to a representative and a clinical sample. Using a meta-heuristic search algorithm, the final reduction to ten items was performed to optimize model fit and discrimination between patients and healthy participants.

#### First item reduction

Factor loadings in the initial item pool ranged between $\lambda$ = 0.30 and $\lambda$ = 0.68. We first deleted 24 items with loadings ≤ 0.60. We additionally identified items clusters for which modification indices indicated different foci of situation type (i.e. romantic relationships, friendships, groups, acquaintances, colleagues) and deleted four items with non-specified situation types to retain a balanced range of specific and general situations within a one-dimensional construct definition. The remaining item pool comprised 18 items which covered a broad range of situations associated with social pain, including a balanced number of specific and general interpersonal situations. Most of the items referred both to emotional responses as well as embedded cognitive aspects, six items focused on emotional responses and one on cognitive aspects (i.e. interpretation of being excluded). Factor loadings of the item pool and selected items can be found in Supplementary Table S1.

#### Second item reduction

Genetic algorithms have increasingly been used for item selection in recent years^26^. Combining elements from random and guided search and allowing for the optimization of multiple, user-defined psychometric criteria regarding the entire item subset, meta-heuristics are powerful algorithms for complex, noisy search spaces^27^. A genetic algorithm was applied to the remaining item pool to optimize the model fit, reliability and discrimination between patients and healthy participants. The best item selection was found in six out of ten replications of the algorithm (see Supplementary Table S1 for item numbers and Supplement S2 for ready to use questionnaire versions in English and German). While the ${\chi }^{2}$-value was significant (${\chi }^{2}$ = 359.06, p < 0.001), the descriptive fit measures now showed an acceptable to good model fit (RMSEA = 0.069, SRMR = 0.029, CFI = 0.970). The composite reliability was excellent (0.940).

### Substudy 3: factorial structure and content validity of the final scale

We cross-validated the factorial structure of the final item selection in a different part of the sample. For convergent and divergent validity, we then examined correlations with related constructs. We calculated group differences between patients and healthy participants, and different diagnostic groups to assess discriminant validity.

#### Measurement invariance and factorial structure

Metric invariance between the normal population and patient sample was supported (${CFI}_{\Delta }$ = 0.001), but not scalar invariance (${CFI}_{\Delta }$ = 0.005). Therefore, it can be assumed that the latent constructs have the same content meaning in the subpopulations studied, but groups should be compared with caution. Based on this model, the ${\chi }^{2}$-value was significant (${\chi }^{2}$ = 335.91, p < 0.001). The RMSEA = 0.076, the CFI = 0.970 and the SRMR = 0.027 indicated an acceptable to good fit. Standardized factor loadings ranged from $\lambda$ = 0.561 to $\lambda$ = 0.856. Detailed results can be found in Supplement S3.

#### Construct validity

Means and standard deviations of all measures can be found in Table 1. For the representative sample, these values can be understood as norm values. However, in the case of the clinical sample, which was not drawn representatively, they can only be understood as preliminary norms. We tested for mean differences between female and male participants. In the clinical sample we found no mean difference (Female: *M* = 2.30, Male: *M* = 2.17, *t* = − 1.14, *p* = 0.259). In the representative sample, there was a significant gender difference found in the Wilcoxon ran sum test (Female: *M* = 1.90, Male: *M* = 1.68, *W* = 691,788, *p* < 0.001).

**Table 1:** Means (or medians) and standard deviations (or interquartile ranges) of measured constructs.

	Sample	N	M/Md	SD/IQR
SPQ (18)^a^	Representative	2531	1.72	1.33
SPQ (10)^a^	Representative	2531	1.80	1.40
SPQ (18)^b^	Clinical	270	2.21	0.79
SPQ (10)^b^	Clinical	270	2.25	0.83
PHQ 4^a^	Representative	2519	1.25	0.75
GBB^a^	Representative	2529	1.38	0.88
BSI-somatization^a^	Clinical	265	0.43	0.86
BSI-interpersonal sensitivity^a^	Clinical	263	1.00	1.5
BSI-depression^a^	Clinical	267	0.83	1.33
BSI-phobix anxiety^a^	Clinical	267	0.40	0.80
BSI-paranoid ideation^a^	Clinical	268	0.60	1.20
IPSM^b^	Clinical	260	2.63	0.42
**BDI-FS**				
SPIN^a^	Online	623	1.19	1.31
MAQ ambivalent-worry^a^	Online	623	2.33	1.67
MAQ ambivalent-merger^a^	Online	623	1.67	1.33

^a^Normality can not be assumed, median and interquartile range are given.

^b^Normality can be assumed, mean and standard deviation are given.

Consistent with our expectations, high correlations (r > 0.50) were observed with the IPSM, the SPIN, and the ambivalent-worry attachment subscale, closely with the BSI Interpersonal Sensitivity subscale (see Table 2). Furthermore, correlations with measures of depression, general psychopathological symptoms and the BSI Paranoid Ideation subscale were moderate. The correlation of the SPQ with the BDI-FS was unexpectedly high. The measures categorized as divergent showed small correlations, except for the unexpected moderate correlation of the SPQ with the BSI Phobic Anxiety subscale.

**Table 2:** Correlations of the 10-item SPQ with convergent and divergent measures.

	Scale	r	Sample	Expectation supportedª
**Expected convergency**				
High	IPSM	0.69**	Clinical	√
	BSI-interpersonal sensitivity	0.47**	Clinical	(√)
	SPIN	0.68**	Online	√
	MAQ ambivalent-worry	0.50**	Online	√
Moderate	BDI-FS	0.64**	Online	(√)
	MAQ ambivalent-merger	0.31**	Online	√
	BSI-depression	0.30**	Clinical	√
	PHQ-4	0.24**	Representative	
	BSI-global severity index	0.34**	Clinical	√
	BSI-paranoid ideation	0.37**	Clinical	√
**Expected divergency**				
	MAQ secure	− 0.06	Online	√
	GBB	0.18**	Representative	√
	BSI-somatization	0.14	Clinical	√
	BSI-phobic anxiety	0.23**	Clinical	

Spearman rank correlations are used because normality could not be assumed for most measures.

*IPSM* interpersonal sensitivity measure, *BSI* brief symptom inventory, *MAQ* measure of attachment qualities, *SPIN* social phobia inventory, *BDI-FS* becks depression inventory—fast screen, *PHQ-4* patient health questionnaire-4.

ªWe expected highly convergent measures to show correlations ≥ 0.50 and moderately convergent measures ≥ 0.30. whereas divergent measures to show correlations ≤ 0.20. **p < 0.001; *p < 0.05.

The construct validity was further supported by the correlation with the 18-item version (*r* = 0.984), showing that the construct is adequately represented in the short version. The 10-item version explained 97.7% of the variance of the 18-item version.

#### Discriminant validity

We found significant differences between the clinical and the representative sample, as well as between patients with high and low rejection-sensitivity diagnoses (Table 3).

**Table 3:** Comparison of SPQ values between representative and clinical sample and between patient subsamples with high or low rejection sensitivity.

Sample/diagnosis	N	M	SD	t	p
Representative sample^a^	2531	1.80	1.40		
Clinical sample	270	2.30	1.10	− 7.46	< 0.001
High rejection-sensitivity Disorders^b^	147	2.38	0.83		
Low rejection-sensitivity disorders^c^	95	2.05	0.76	− 3.21	0.002

^a^SPQ for the representative sample is not normally distributed. Median and interquartile range are given, and Yuan-Bentler test is reported.

^b^Personality disorders, depression, social anxiety disorder.

^c^Other anxiety disorders, trauma-related disorders.

A ROC analysis revealed poor discrimination between patients and healthy participants (AUC = 0.634). The cut-off value of 1.83 showed a sensitivity of 0.678 and a specificity of 0.507 and classified 52.3% of participants accurately. The discriminatory power was higher for rejection-sensitive patients versus other participants (AUC = 0.670). The determined cut-point (SPQ = 2.16) classified 64.1% of the participants accurately (sensitivity = 0.640; specificity = 0.641).

## Discussion

The aim of the studies was to develop and validate a brief self-report instrument assessing emotional reactions of individuals to social exclusion, rejection and relational devaluation. Based on the background of the social-physical pain overlap theory^4^ and belongingness theory^1^, an initial pool was shortened in a two-step procedure, based on a total sample of 3424 respondents, to a final version with 10 items. We found that the final SPQ was associated with a solid one-factor-structure and excellent reliability.

To establish construct validity, we used rating scales of related constructs to demonstrate convergent validity. Most closely related to the target construct, the Interpersonal Sensitivity Measure^22^, which measures perception or anticipation of criticism or rejection by others, demonstrated the highest correlation. A high correlation was also found with the BSI-subscale Interpersonal Sensitivity, which assesses feelings of personal inadequacy and inferiority^28^, reflecting most evidently the convergent validity of the SPQ. Furthermore, as predicted by the interpersonal theory of Leary^5^, we also found significant, although moderate correlations to anxious-ambivalent attachment. Thus, individuals high on social pain might be characterized by a high need for intimacy on one side, and a high level of neuroticism and low level of openness to experience on the other side^29^.

In addition, given the close theoretical relationship to social pain, we expected moderate to high correlations with measures of depression^11,13^ and social anxiety^16,30^. These expectations were confirmed, in particular for depression scales which cover not only core symptoms of depression but also interpersonal problems. Interestingly, the high correlation with the BSI-subscale Paranoid Ideation indicates that high SPQ values may also reflect disordered thinking like projective thoughts and suspiciousness in transition to more severe psychiatric disorders, such as paranoid delusions.

We also tested the discriminant validity of the SPQ by comparing clinical subgroups with diagnoses related to varying degrees to social pain. In line with our expectations, patients with depression, SAD and personality disorders showed higher levels of social pain than patients with other diagnoses (anxiety disorders other than SAD, trauma-related disorders). In addition, our clinical sample showed significantly elevated scores in SPQ compared to the representative sample. Interestingly, the scores were normally distributed in the clinical sample showed stronger skewness in the normal population (clinical sample = − 0.05, representative sample = 0.09). In the representative sample a considerable number of participants (n = 113) had a score of exactly 0, which was not true for the clinical sample. This supports the subclinical nature of the construct. To identify patients with increased social pain for specific psychological interventions, we determined preliminary cut-off values based on the comparisons of the clinical sample with the representative sample, and of patients with rejection-sensitive diagnoses with clinical and non-clinical controls. However, the identification of patients with high sensitivity for rejection allowed for a higher rate of correct classification (64.1%) than individuals with mental disorders (52.3%). Thus, it seems to be more appropriate to use the SPQ cut-off to identify patients with high individual susceptibility to social pain, rather than for the identification of mental disorders.

The present study has several limitations.

First, we did not involve expert ratings on the relevance of the items in the development of the item pool, which would have increased content validity^31^. However, the questionnaire was developed in German and no experts were available for content ratings. Therefore, we strongly based the definition of items on theoretical considerations, deriving a predefined set of situational variables and emotional reactions reported from literature.

Second, for the demonstration of convergent validity, although we defined apriori criteria for convergency and used descriptive analyses of the size of correlations, data from other sources such as cyberball experiments may be more conclusive.

Third, we included samples which were recruited in different ways. The online survey was a convenience sample, and self-selection may have influenced the characteristics of the sample. Furthermore, the clinical sample was much smaller than the others, with limited options to create sufficiently large diagnostic subsamples. Thus, also the SPQ cut-off value we obtained here is only preliminary.

Fourth, the test–retest reliability of the SPQ was not examined, which needs to be addressed in future studies.

Fifth, due to the lack of latent measurement invariance, group comparisons should be interpreted with care. Future studies are needed to investigate the causes of the measurement invariance problems. For now, the scale seems to be more appropriate for comparisons between patients.

Finally, the data-driven selection approach poses the risk of over-fitting, but we mitigated this problem by reserving a portion of the sample for independent cross-validation.

In conclusion, apart from these limitations, the SPQ in this study was associated with robust psychometric properties with regard to factor structure, reliability, and convergent and discriminant validity, which supports its use in clinical and nonclinical populations and may close a gap in the assessment of social pain. It should be noted that the process of item selection favored items referring not to specific persons, such as family members or partners. However, since the SPQ is primarily designed to screen for social pain in clinical populations, therapists and counsellors should explore in the individual case more details about the triggers and persons associated with social pain. The next phase of research will include experimental studies to investigate the prediction of emotional reactions in cyberball experiments with clinical samples of depressed and socially anxious patients^30^. Furthermore, it will be interesting to tailor interventions such as cognitive restructuring, behavioral experiments, or acceptance and commitment therapy to the needs of patients with high social pain^32^, and to assess treatment sensitivity in future intervention trials.

## Methods and measures

### Sample

We recruited three samples during the study. All methods were carried out in accordance with relevant guidelines and regulations. Study 1 and Study 3 were approved by the research ethics board of the Department of Psychology, Goethe University Frankfurt, and Study 2 was approved the ethics board of the Department of Medicine, University of Leipzig. Informed consent was obtained from all subjects or, if subjects are under 18, from a parent and/or legal guardian. No compensation was given for participation.

The first sample was recruited via the internet, local newspapers, and flyers. The only exclusion criterion was a minimum age of 17 years. The sample comprised 623 individuals with a mean age of 32.61 (*SD* = 11.13). 39% of the participants reported a mental disorder, most frequently depression (22.8%).

The second sample was recruited in a national, representative general population survey. The data were collected in 2 waves between May and June 2019 by professional demographic consultants (Unabhängige Serviceeinrichtung für Umfragen, Methoden und Analysen, Berlin). 2531 households were eligible to participate and were visited by trained face-to-face interviewers who recorded participants’ demographic information; other information was collected through a paper/pencil self-report.

Data for the third sample were collected between June 2018 and October 2019. Patients who were admitted to psychotherapy at the Center for Cognitive Behavioral Therapy of the Goethe University Frankfurt were informed about the goals of the study. Inclusion criteria comprised at least one diagnosis of mental disorder according to ICD-10, and an age of 18 years or more. The participation was voluntary and not part of the treatment. Participants were invited to complete the questionnaires on PC before starting treatment. Sociodemographic characteristics for the online sample (*n* = 623), the representative sample (*n* = 2531) and the patient sample (*n* = 270) are given in Table 4.

**Table 4:** Sociodemographic characteristics of the three samples.

	Online N = 623	Representative N = 2531	Clinical N = 270
**Age (years)**			
M (SD)	32.61 (11.13)	48.44 (17.86)	37.23 (12.79)
Range	17–66	17–95	19–74
**Gender**			
Female		64,0%	62.6%
Male		46.0%	37.4%
**Marital status**			
Single	41.40%	30.0%	42.5%
Firm partnership	31.30%	31.30%	20.4%
Married	19.90%	43.5%	26.6%
Divorced	7.40%	14.5%	8.7%
**Education**			
No degree	1.80%	2.4%	3.7%
Elementary school	7.40%	27.2%	11.2%
High school diploma	19.30%	39.8%	18.5%
Bachelor degree	35.30%	17.4%	34.1%
Master degree	36.30%	9.9%	25.9%
Not specified		3.3%	6.6%
**Employment**			
Employed	45.60%	59.3%	73.7%
Unemployed	7.90%	4.1%	3.0%
Homemaker	3.50%	3.4%	2.6%
Student/school	32.90%	6.8%	13.4%
In training	4.30%	1.7%	2.1%
Retired	5.80%	24.5%	5.1%
**Primary diagnoses**			
Depression			31.1%
Social anxiety disorder			14.9%
Other anxiety disorders			17.3%
Trauma-related disorders			19.6%
Personality disorder			10.0%
Other diagnoses			6.3%

### Statistical analysis

Normality for all measures was tested using the Shapiro–Wilk test and inspection of Q–Q plots. The SPQ-10 test scores were normally distributed in the patient sample (*W* = 0.99, *p* = 0.14), but not in the representative sample (*W* = 0.98, *p* < 0.001). Normal distribution could not be assumed for several of the measures, especially clinical and subclinical measures, which is consistent with theory. Therefore, we use nonparametric methods where necessary, e.g. Spearman rank correlation, robust linear regression etc.

The factorial structure and model fit of the questionnaire were checked at several stages by means of confirmatory factor analysis (CFA), using the R-package *lavaan*^33^. Since normality could not be assumed in all cases, we used the robust maximum likelihood estimator (MLR), that provides more accurate results than maximum likelihood estimation^34^. In addition to the ${\chi }^{2}$-test, we used the root mean square error of approximation (RMSEA) and the standardized root mean square residual (SRMR) as assessment for the absolute fit and the comparative fit index (CFI) for the relative fit. RMSEA and SRMR values of < 0.05 and < 0.08; and a CFI of > 0.97 and > 0.95 are considered as good and acceptable, respectively^35^.

For the first item reduction, items with a loading of $\lambda$ > 0.60 were selected. The final selection was carried out using a genetic algorithm (GA)^36^ implemented in the R-package *stuart*^37^. Within the *stuart*-framework, all parameters derived from CFA can be included in the objective function. In this study, we optimized RMSEA and SRMR, model-based reliability and discrimination between patients and healthy participants. Since meta-heuristics solve optimization problems probabilistically, the algorithm was applied ten times to ensure reliability. Additional details regarding the application of the genetic algorithm can be found online in Supplement S4. The full sample was randomly split into a *construction* (*n* = 1401) and a *validation sample* (*n* = 1400) for unbiased cross-validation.

Measurement invariance between the representative and the clinical sample was evaluated using a stepwise procedure^38^ with a cut-off value of ΔCFI $\ge$ 0.005^39^.

To assess convergent and divergent validity, Spearman rank correlations with measures of convergent (interpersonal sensitivity, attachment styles, depression and social anxiety) and divergent (somatic complaints) dimensions were calculated from the three samples.

To assess discriminant validity, patients with primary diagnoses of personality disorders, depression and social anxiety disorders (*n* = 147), empirically related to rejection sensitivity or social pain, were compared to patients with other anxiety disorders or trauma-related disorders (*n* = 95). The Yuan–Bentler test was used for comparisons where normality could not be assumed. A ROC analysis was conducted to determine the discriminatory power for the discrimination of patients from healthy participants, and of patients with rejection-sensitive diagnoses from all other participants. A cut-off point was calculated by maximizing the sum of sensitivity and specificity.

All statistical tests were conducted with $\alpha$ = 0.05. Analyses were conducted with the R statistical environment, using the packages *robustbase*^40^, *pRoc*^41^ and *cutpointr*^42^.

### Measures

The *Social Phobia Inventory* (SPIN)^43^ is a 17-item screening instrument which evaluates fear, avoidance, and physiological discomfort in a variety of social situations. The internal consistency (Cronbach’s α = 0.82–0.95, here $\omega$ = 0.94) and convergent and divergent validity^43–45^.

The *Measure of Attachment Qualities* (MAQ)^46^ is a 14-item self-report measure of adult attachment patterns. It comprises subscales of secure and avoidant attachment, ambivalent-worrying and ambivalent/merged. Internal consistency was moderate (Cronbach’s α = 0.70). Individuals high in anxious attachment tend to be sensitive to rejection cues^47^.

The *Patient Health Questionnaire-4* (PHQ-4)^48^ is a brief screening scale for anxiety and depression. Four items refer to common symptoms of anxiety and depression. The PHQ-4 has been validated in clinical^48^ and general population^49^ samples.

The *Somatic Complaints Inventory* (GBB)^50^ was used to assess physical well-being. Eight items refer to somatic complaints. In large epidemiological samples, reliability, item characteristics, and factor structure of the GBB were excellent^51^, here internal consistency was high ($\omega$ = 0.89).

The *Brief Symptom Inventory* (BSI)^28^ is a 53-item-self-report instrument to assess psychological symptoms. It is composed of nine primary symptom dimensions. The total score (Global Severity Index) measures the overall psychological distress. In psychiatric patients, internal consistencies (α = 0.71–0.85; present study: $\omega$ = 0.73 to 0.85) and test–retest reliabilities (${r}_{tt} =$ 0.68–0.91) of the subscales are high. The BSI has shown a high convergent, discriminant, and construct validity in clinical samples^52^.

The *Interpersonal Sensitivity Measure* (IPSM)^22^ is a 36-item self-report instrument assessing perception or anticipation of criticism and rejection. The instrument proved as reliable (here $\omega$ = 0.91) and valid in predicting the development of depressive episodes and low self-esteem^53^, social anxiety disorder^24^, and persecutory ideations^54^.

#### Beck depression inventory-fast screen (BDI-FS; Beck et al.^55^)

The BDI-FS is a seven-item self-report inventory designed to evaluate depression in patients with medical illness. It has been proven a reliable and valid measure of depression in the German general population^56^, with an internal consistency of α = 0.84.

## Supplementary Information

Supplementary Information 1.

Supplementary Information 2.

## Data Availability

The datasets generated during and/or analysed during the current study are available in the OSF repository (https://osf.io/m78sx/?).

## Code availability

The full code of the genetic algorithm application is available in the OSF repository (https://osf.io/m78sx/?).
