# Supplementary material for: Development and validation of a new instrument to measure social pain
Source: Sci Rep. 2021 Apr 15;11:8283. doi: 10.1038/s41598-021-87351-3 (PMC8050222; doi:10.1038/s41598-021-87351-3)
Supplement: Supplementary file 1 — Supplementary Information 1. [file 41598_2021_87351_MOESM1_ESM.docx]

**Appendix**

**Final version of the Social Pain Questionnaire (SPQ)**

(Items translated from German into English^[[1]](#footnote-1)^ )

| English Version | German Version |
| --- | --- |
| 1. It hurts my feelings if somebody denies a request of me. | 1. Wenn mir jemand eine Bitte abschlägt, verletzt das meine Gefühle. |
| 2. I feel very humiliated when I am excluded from a group. | 2. Wenn ich durch eine Gruppe ausgeschlossen werde, kränkt mich das sehr. |
| 3. I feel insulted when being ignored at a party. | 3. Wenn mich auf einer Feier ein Bekannter nicht beachtet, kränkt mich das. |
| 4. It hurts me when somebody ignores me. | 4. Wenn mich jemand ignoriert, verletzt mich das sehr. |
| 5. When I feel rejected, I experience inner tension. | 5. Wenn ich mich zurückgewiesen fühle, nehme ich eine innere Anspannung wahr. |
| 6. When an acquaintance does not respond to me when I say hello, I feel rejected. | 6. Wenn mich ein Bekannter nicht zurück grüßt, fühle ich mich abgelehnt. |
| 7. When a friend distances himself/herself from me, I feel repulsed. | 7. Wenn sich ein Freund von mir distanziert, fühle ich mich stark zurückgewiesen. |
| 8. When I get the impression that a colleague withdraws from me, I feel rejected. | 8. Wenn ich das Gefühl habe, dass sich ein Kollege von mir zurückzieht, fühle ich mich abgelehnt. |
| 9. When somebody declines my request or suggestion, I feel snubbed. | 9. Wenn mir jemand eine Abfuhr erteilt, fühle ich mich vor den Kopf gestoßen. |
| 10. If somebody cancels an appointment without a good reason, I feel repulsed. | 10. Wenn jemand ohne einen triftigen Grund ein Treffen absagt, fühle ich mich zurückgewiesen. |

1. Translation process: The instrument was first translated from its original German version into English by an American academic psychologist living in Germany (Dr. Adriane Cavallini, Frankfurt University). The English version was then back-translated into German by an American psychologists born in Germany with wide experience in the development of clinical scales (Dr. Stefan G. Hofmann, Boston University). The two versions were compared and discrepancies discussed by the two bilingual raters and the first author of this article to verify the semantic equivalence of the English translated version of the questionnaire. The final English version is based on the consensus of all three raters. [↑](#footnote-ref-1)
