# Supplementary material for: Development and validation of a new instrument to measure social pain
Source: Sci Rep. 2021 Apr 15;11:8283. doi: 10.1038/s41598-021-87351-3 (PMC8050222; doi:10.1038/s41598-021-87351-3)
Supplement: Supplementary file 2 — Supplementary Information 2. [file 41598_2021_87351_MOESM2_ESM.docx]

**Development and validation of a new instrument to measure social pain**

Ulrich Stangier1, Dr.*, Johanna Schüller1, M.Sc. & Elmar Brähler2, Dr.

**Supplementary Material**

**Supplement S1**

| **Supplementary Table S1.** *Overview of factor loadings in the inital item pool and selected items for 18- and 10-item versions* | | | |  | **Supplementary Table S1 continued.** | | | |
| --- | --- | --- | --- | --- | --- | --- | --- | --- |
| 46-Item-Version | 𝜆 | 18-Item-Version | 10-Item-Version |  | 46-Item-Version | 𝜆 | 18-Item-Version | 10-Item-Version |
| 1 | .60 | 1 |  |  | 24 | .57 |  |  |
| 2 | .60 | 2 | 1 |  | 25 | .30 |  |  |
| 3 | .49 |  |  |  | 26 | .61 | 12 | 7 |
| 4 | .50 |  |  |  | 27 | .55 |  |  |
| 5 | .60 | 3 | 2 |  | 28 | .60 |  |  |
| 6 | .53 |  |  |  | 29 | .61 |  |  |
| 7 | .61 | 4 |  |  | 30 | .59 |  |  |
| 8 | .64 | 5 |  |  | 31 | .58 |  |  |
| 9 | .66 | 6 |  |  | 32 | .64 | 13 |  |
| 10 | .55 |  |  |  | 33 | .43 |  |  |
| 11 | .67 | 7 | 3 |  | 34 | .68 | 14 | 8 |
| 12 | .54 | 8 |  |  | 35 | .62 | 15 | 9 |
| 13 | .61 |  |  |  | 36 | .36 |  |  |
| 14 | .58 |  |  |  | 37 | .42 |  |  |
| 15 | .53 |  |  |  | 38 | .55 |  |  |
| 16 | .52 |  |  |  | 39 | .41 |  |  |
| 17 | .41 |  |  |  | 40 | .49 |  |  |
| 18 | .60 | 9 | 5 |  | 41 | .62 | 16 |  |
| 19 | .59 |  |  |  | 42 | .44 |  |  |
| 20 | .66 | 10 | 6 |  | 43 | .51 |  |  |
| 21 | .39 |  |  |  | 44 | .65 | 17 |  |
| 22 | .57 |  |  |  | 45 | .63 | 18 | 10 |
| 23 | .66 | 11 |  |  | 46 | .60 |  |  |

**Supplement S2**

**Social Pain Questionnaire - English version^[[1]](#footnote-1)^**

*The following questionnaire refers to emotional reactions on social situations. Please indicate for each of the following statements, to which extent these apply to you. There are no right or wrong answers. Please mark for each of the statements only one box.*

|  | |  | Applies **exactly**  to me | Applies **largely**  to me | Applies  **in part**  to me | Applies **rather not**  to me | Applies **not at all** to me |
| --- | --- | --- | --- | --- | --- | --- | --- |
|  | |  |  |  |  |  |  |
| 1 | It hurts my feelings if somebody denies a request of me. | |  |  |  |  |  |
| 2 | I feel very humiliated when I am excluded from a group. | |  |  |  |  |  |
| 3 | I feel insulted when being ignored at a party. | |  |  |  |  |  |
| 4 | It hurts me when somebody ignores me. | |  |  |  |  |  |
| 5 | When I feel rejected, I experience inner tension. | |  |  |  |  |  |
| 6 | When an acquaintance does not respond to me when I say hello, I feel rejected. | |  |  |  |  |  |
| 7 | When a friend distances himself/herself from me, I feel repulsed. | |  |  |  |  |  |
| 8 | When I get the impression that a colleague withdraws from me, I feel rejected. | |  |  |  |  |  |
| 9 | When somebody declines my request or suggestion, I feel snubbed. | |  |  |  |  |  |
| 10 | If somebody cancels an appointment without a good reason, I feel repulsed. | |  |  |  |  |  |

**Social Pain Questinnaire - German version**

**Fragebogen zu sozialem Schmerz**

*Die nachfolgenden Fragen beziehen sich auf Ihre emotionalen Reaktionen auf soziale Situationen. Bitte geben Sie für jede der folgenden Aussagen an, wie sehr diese auf Sie zutrifft. Es gibt keine richtigen oder falschen Antworten. Kreuzen Sie bei* ***jeder*** *Aussage immer nur* ***ein*** *Kästchen an.*

|  | |  | Trifft  **genau** zu | Trifft **eher** zu | Trifft **teilweise** zu | Trifft **eher nicht** zu | Trifft **gar nicht** zu |
| --- | --- | --- | --- | --- | --- | --- | --- |
|  | |  |  |  |  |  |  |
| 1 | Wenn mir jemand eine Bitte abschlägt, verletzt das meine Gefühle. | |  |  |  |  |  |
| 2 | Wenn ich durch eine Gruppe ausgeschlossen werde, kränkt mich das sehr. | |  |  |  |  |  |
| 3 | Wenn mich auf einer Feier ein Bekannter nicht beachtet, kränkt mich das. | |  |  |  |  |  |
| 4 | Wenn mich jemand ignoriert, verletzt mich das sehr. | |  |  |  |  |  |
| 5 | Wenn ich mich zurückgewiesen fühle, nehme ich eine innere Anspannung wahr. | |  |  |  |  |  |
| 6 | Wenn mich ein Bekannter nicht zurück grüßt, fühle ich mich abgelehnt. | |  |  |  |  |  |
| 7 | Wenn sich ein Freund von mir distanziert, fühle ich mich stark zurückgewiesen. | |  |  |  |  |  |
| 8 | Wenn ich das Gefühl habe, dass sich ein Kollege von mir zurückzieht, fühle ich mich abgelehnt. | |  |  |  |  |  |
| 9 | Wenn mir jemand eine Abfuhr erteilt, fühle ich mich vor den Kopf gestoßen. | |  |  |  |  |  |
| 10 | Wenn jemand ohne einen triftigen Grund ein Treffen absagt, fühle ich mich zurückgewiesen. | |  |  |  |  |  |

**Supplement S3**

**Supplementary Table S3a.**

*Model fit indices for the different levels of measurement invariance, and* 𝜒2*-difference test.*

| Invariance Level | 𝜒2 | df | p | CFI | RMSEA | SRMR | ${\chi^{2}}_{\Delta}$ | $p_{\Delta}$ |
| --- | --- | --- | --- | --- | --- | --- | --- | --- |
| Configural | 319.91 | 70 | < .001 | .970 | .080 | .025 |  |  |
| Metric | 335.91 | 79 | < .001 | .970 | .076 | .027 | 8.41 | .494 |
| Scalar | 405.73 | 88 | < .001 | .963 | .079 | .032 | 74.88 | < .001 |
| *Note.* Model-fit indices from confirmatory analysis using a robust maximum likelihood estimation (MLR). Configural invariance: no parameters equated between groups. Metric invariance: Factor loadings equated across groups. Scalar invariance: Item intercepts equated across groups. | | | | | | | | |

**Supplementary Table S3b.**

*Standardized factor loadings from the validation sample in the representative and the patient group.*

| Item Nr. | 𝜆 (Representative sample) | 𝜆 (Patient sample) |
| --- | --- | --- |
| 2 | .758 | .647 |
| 3 | .779 | .679 |
| 5 | .847 | .744 |
| 7 | .856 | .746 |
| 9 | .797 | .645 |
| 10 | .833 | .683 |
| 12 | .781 | .684 |
| 14 | .798 | .674 |
| 15 | .849 | .754 |
| 18 | .762 | .561 |
| *Note.* Loadings are standardized for interpretability. Factor loadings between both groups are equated, but loadings differ through the standardization. | | |

**Supplement S4**

**Details regarding the application of the genetic algorithm**

The objective function used here is given by

$$f = \frac{1}{1 + e^{4-10*Rel}} + 0.5 * (1- \frac{1}{1+e^{5-100*RMSEA}}) + 0.5 * (1-\frac{1}{1+e^{5-100*SRMR}}) + \frac{1}{1+e^{5-10*({MW}_{diff} + 0.5)}},$$

with *Rel* as model-based composite reliability, calculated as the amount of true score variance relative to the total amount of variance. For the optimization of the model fit, we included RMSEA and SRMR. In addition, the mean difference between patients and healthy controls was maximized. Following the default settings of the stuart package (Package version 0.9.0), the number of individuals is set to 64, the reproduction rate is at 50 % and the maximum number of generations is set to 256. The algorithm is reinitialized once, when the reinitialization criterion is reached. Chromosomes are mated with the most dissimilar individuals in each round, to increase exploration of the search space. The algorithm is terminated, when the change in selection probabilities undercuts the tolerance value .01 in three subsequent generations. The algorithm is executed 10 times, with the following, randomly generated seeds for reproducibility: 92404 49321 88689 62995 93420 69684 72033 60387 90470 71305.

**Suppement S5**

| **Supplementary Table S5a.**  *Norms for the representative sample* | | | | | | | |
| --- | --- | --- | --- | --- | --- | --- | --- |
| Female | | | | Male | | | |
| Raw score | z | T | PR | z | T | PR | Raw score |
| 0 | -1.99 | 30 | 0 | -1.64 | 34 | 0 | 0 |
| 1 | -1.88 | 31 | 3 | -1.54 | 35 | 6 | 1 |
| 2 | -1.78 | 32 | 4 | -1.44 | 36 | 8 | 2 |
| 3 | -1.67 | 33 | 5 | -1.35 | 36 | 11 | 3 |
| 4 | -1.57 | 34 | 6 | -1.25 | 38 | 13 | 4 |
| 5 | -1.46 | 35 | 8 | -1.15 | 38 | 15 | 5 |
| 6 | -1.36 | 36 | 10 | -1.05 | 40 | 17 | 6 |
| 7 | -1.25 | 38 | 11 | -0.96 | 40 | 19 | 7 |
| 8 | -1.15 | 38 | 13 | -0.86 | 41 | 20 | 8 |
| 9 | -1.04 | 40 | 15 | -0.76 | 42 | 23 | 9 |
| 10 | -0.94 | 41 | 17 | -0.66 | 43 | 25 | 10 |
| 11 | -0.83 | 42 | 20 | -0.57 | 44 | 29 | 11 |
| 12 | -0.73 | 43 | 23 | -0.47 | 45 | 32 | 12 |
| 13 | -0.62 | 44 | 26 | -0.37 | 46 | 35 | 13 |
| 14 | -0.52 | 45 | 30 | -0.27 | 47 | 38 | 14 |
| 15 | -0.42 | 46 | 33 | -0.18 | 48 | 42 | 15 |
| 16 | -0.31 | 47 | 37 | -0.08 | 49 | 45 | 16 |
| 17 | -0.21 | 48 | 40 | 0.02 | 50 | 49 | 17 |
| 18 | -0.10 | 49 | 43 | 0.12 | 51 | 53 | 18 |
| 19 | 0.00 | 50 | 46 | 0.22 | 52 | 56 | 19 |
| 20 | 0.11 | 51 | 51 | 0.31 | 53 | 60 | 20 |
| 21 | 0.21 | 52 | 56 | 0.41 | 54 | 66 | 21 |
| 22 | 0.32 | 53 | 60 | 0.51 | 55 | 70 | 22 |
| 23 | 0.42 | 54 | 65 | 0.61 | 56 | 73 | 23 |
| 24 | 0.53 | 55 | 68 | 0.70 | 57 | 76 | 24 |
| 25 | 0.63 | 56 | 71 | 0.80 | 58 | 78 | 25 |
| 26 | 0.74 | 57 | 74 | 0.90 | 59 | 80 | 26 |
| 27 | 0.84 | 58 | 77 | 1.00 | 60 | 82 | 27 |
| 28 | 0.95 | 60 | 80 | 1.09 | 61 | 84 | 28 |
| 29 | 1.05 | 60 | 83 | 1.19 | 62 | 85 | 29 |
| 30 | 1.16 | 62 | 85 | 1.29 | 63 | 87 | 30 |
| 31 | 1.26 | 63 | 88 | 1.39 | 64 | 89 | 31 |
| 32 | 1.36 | 64 | 90 | 1.48 | 65 | 91 | 32 |
| 33 | 1.47 | 65 | 92 | 1.58 | 66 | 93 | 33 |
| 34 | 1.57 | 66 | 93 | 1.68 | 67 | 93 | 34 |
| 35 | 1.68 | 67 | 94 | 1.78 | 68 | 94 | 35 |
| 36 | 1.78 | 68 | 95 | 1.87 | 69 | 95 | 36 |
| 37 | 1.89 | 69 | 97 | 1.97 | 70 | 96 | 37 |
| 38 | 1.99 | 70 | 97 | 2.07 | 71 | 97 | 38 |
| 39 | 2.10 | 71 | 98 | 2.17 | 72 | 97 | 39 |
| 40 | 2.20 | 72 | 98 | 2.26 | 73 | 98 | 40 |

| **Supplementary Table S5b.**  *Preliminary norm values for the clinical sample* | | | | | | | | |
| --- | --- | --- | --- | --- | --- | --- | --- | --- |
| Female | | | | Male | | | | |
| Raw score | z | T | PR | z | T | PR | Raw score |  |
| 0 | -2.80 | 22 | 0 | -2.54 | 25 | 0 | 0 |  |
| 1 | -2.67 | 23 | 0 | -2.42 | 26 | 0 | 1 |  |
| 2 | -2.55 | 24 | 1 | -2.31 | 27 | 0 | 2 |  |
| 3 | -2.43 | 26 | 1 | -2.19 | 28 | 0 | 3 |  |
| 4 | -2.31 | 27 | 1 | -2.07 | 29 | 0 | 4 |  |
| 5 | -2.18 | 28 | 1 | -1.95 | 30 | 3 | 5 |  |
| 6 | -2.06 | 29 | 1 | -1.84 | 32 | 3 | 6 |  |
| 7 | -1.94 | 31 | 1 | -1.72 | 33 | 7 | 7 |  |
| 8 | -1.82 | 32 | 2 | -1.60 | 34 | 8 | 8 |  |
| 9 | -1.70 | 33 | 4 | -1.49 | 35 | 8 | 9 |  |
| 10 | -1.57 | 34 | 5 | -1.37 | 36 | 9 | 10 |  |
| 11 | -1.45 | 36 | 6 | -1.25 | 38 | 11 | 11 |  |
| 12 | -1.33 | 37 | 8 | -1.13 | 39 | 12 | 12 |  |
| 13 | -1.21 | 38 | 9 | -1.02 | 40 | 15 | 13 |  |
| 14 | -1.09 | 39 | 13 | -0.90 | 41 | 17 | 14 |  |
| 15 | -0.96 | 40 | 15 | -0.78 | 42 | 21 | 15 |  |
| 16 | -0.84 | 42 | 18 | -0.67 | 43 | 25 | 16 |  |
| 17 | -0.72 | 43 | 24 | -0.55 | 44 | 27 | 17 |  |
| 18 | -0.60 | 44 | 27 | -0.43 | 46 | 30 | 18 |  |
| 19 | -0.48 | 45 | 30 | -0.31 | 47 | 36 | 19 |  |
| 20 | -0.35 | 46 | 34 | -0.20 | 48 | 39 | 20 |  |
| 21 | -0.23 | 48 | 40 | -0.08 | 49 | 43 | 21 |  |
| 22 | -0.11 | 49 | 44 | 0.04 | 50 | 49 | 22 |  |
| 23 | 0.01 | 50 | 48 | 0.15 | 52 | 52 | 23 |  |
| 24 | 0.13 | 51 | 55 | 0.27 | 53 | 56 | 24 |  |
| 25 | 0.26 | 53 | 62 | 0.39 | 54 | 58 | 25 |  |
| 26 | 0.38 | 54 | 66 | 0.50 | 55 | 62 | 26 |  |
| 27 | 0.50 | 55 | 70 | 0.62 | 56 | 68 | 27 |  |
| 28 | 0.62 | 56 | 73 | 0.74 | 57 | 76 | 28 |  |
| 29 | 0.74 | 57 | 74 | 0.86 | 59 | 80 | 29 |  |
| 30 | 0.87 | 59 | 77 | 0.97 | 60 | 82 | 30 |  |
| 31 | 0.99 | 60 | 79 | 1.09 | 61 | 86 | 31 |  |
| 32 | 1.11 | 61 | 82 | 1.21 | 62 | 88 | 32 |  |
| 33 | 1.23 | 62 | 85 | 1.32 | 63 | 90 | 33 |  |
| 34 | 1.35 | 64 | 88 | 1.44 | 64 | 92 | 34 |  |
| 35 | 1.48 | 65 | 92 | 1.56 | 66 | 94 | 35 |  |
| 36 | 1.60 | 66 | 93 | 1.68 | 67 | 95 | 36 |  |
| 37 | 1.72 | 67 | 93 | 1.79 | 68 | 96 | 37 |  |
| 38 | 1.84 | 68 | 95 | 1.91 | 69 | 97 | 38 |  |
| 39 | 1.97 | 70 | 96 | 2.03 | 70 | 98 | 39 |  |
| 40 | 2.09 | 71 | 99 | 2.14 | 71 | 98 | 40 |  |

1. Translation process: The instrument was first translated from its original German version into English by an American academic psychologist living in Germany (Dr. Adriane Cavallini, Frankfurt University). The English version was then backtranslated into German by an American psychologists with wide experience in the development of clinical scales born in Germany (Dr. Stefan G. Hofmann, Boston University). The two versions were compared and discrepancies discussed by the two bilingual raters and the first author of this article to verify the semantic equivalence of the English translated version of the questionnaire. The final English version is based on the consensus of all three raters. [↑](#footnote-ref-1)
